# Fecal microbiota transplantation alleviated heat-induced colonic tissue damage, epithelial apoptosis, and oxidative stress

**DOI:** 10.1128/aem.00976-25

**Published:** 2025-09-24

**Authors:** Xiyu Liu, Chuchu Liu, Xiaoli Qian, Shiqing Zhang, Zhenghong Yao, Yanxi Chai, Qianhan Shi, Wenwen Yang, Qingxian Wang, Lina Zhang, Xiang Zeng, Cuiqing Liu, Yue Wu, Qinghua Sun

**Affiliations:** 1 School of Public Health, Zhejiang Chinese Medical University 70571, Hangzhou, Zhejiang, China; 2 International Science and Technology Cooperation Base of Air Pollution and Health, Hangzhou, Zhejiang, China; University of Illinois Urbana-Champaign, Urbana, Illinois, USA

**Keywords:** heat exposure, fecal microbiota transplantation, colonic damage, apoptosis, gut microbiota, oxidative stress

## Abstract

Exposure to high ambient temperatures can cause significant damage to the gastrointestinal tract; however, the therapeutic potential of fecal microbiota transplantation (FMT) in this context remains largely unexplored. We investigated whether FMT could alleviate heat-induced colonic injury in C57BL/6J mice. Mice were randomly divided into four groups: normal control (22°C only), normal-FMT (NF, 22 °C+ FMT), heat exposure (HE, 39°C only), and HE-FMT (HF, 39°C + FMT). The HE and HF groups were exposed to 39°C for 2 hours daily over 15 consecutive days. FMT (100 µL/day) was administered by oral gavage to the NF and HF groups for 15 days, starting after the first HE. Our results showed that FMT significantly modulated gut microbiota composition, increasing the relative abundance of *Alistipes*, *Citrobacter*, *Parasutterella*, *Bifidobacterium, Lachnospiraceae_UCG-001*, *Raoultella*, *Woeseia*, *Prevotellaceae_UCG-001,* and *Christensenellaceae,* while decreasing *Clostridium_sensu_stricto_1*, *Eubacterium_xylanophilum_group*, *Clostridioides*, *Bilophila*, *GCA-900066575,* and *Peptococcus*. Notably, FMT markedly restored epithelial integrity and enhanced mucus production, as shown by hematoxylin-eosin and periodic acid-Schiff staining. Moreover, FMT attenuated heat-induced epithelial cell apoptosis, evidenced by reduced apoptotic cells and downregulation of mitochondrial apoptotic markers, including Bax, Bak, cleaved Caspase-3, cleaved Caspase-9, and the phospho-P53/P53 ratio. In addition, FMT mitigated oxidative stress induced by HE, indicated by decreased 3-nitrotyrosine levels and normalization of antioxidant-related proteins, such as *Nrf2*, *Sod1*, *Cat*, and *Gpx4*. Collectively, these findings demonstrate that FMT alleviates heat-induced colonic injury by restoring mucosal barrier integrity, inhibiting apoptosis, and reducing oxidative stress, highlighting its potential as a promising therapeutic strategy for heat-related gastrointestinal disorders.

**IMPORTANCE:**

This study is the first to demonstrate the protective role of fecal microbiota transplantation (FMT) against heat-induced colonic injury in a mouse model. We show that FMT mitigates colonic damage by restoring gut microbiota balance, preserving mucosal barrier integrity, inhibiting epithelial cell apoptosis, and reducing oxidative stress. These findings underscore the essential role of the gut microbiota in maintaining intestinal homeostasis under heat stress and highlight the therapeutic potential of microbiota-targeted strategies, such as FMT, in preventing or treating heat-related intestinal injury.

## INTRODUCTION

Over the past century, global temperatures have risen by approximately 1.1°C since pre-industrial times, accompanied by an increasing frequency of extreme weather events such as heatwaves (1–3). This persistent warming trend poses a significant threat to human health, contributing to heat-related illnesses, including heat exhaustion and heat stroke, and is linked to higher rates of mortality, emergency department visits, and hospitalizations for cardiovascular and respiratory diseases (4–7). According to the World Health Organization, the number of individuals exposed to extreme heat is rising rapidly (8). The 2019 Global Burden of Disease study estimated that high temperatures were responsible for approximately 0.31 million deaths and 11.70 million disability-adjusted life years (DALYs) globally, accounting for 0.54% of total deaths and 0.46% of total DALYs (9).

Beyond cardiovascular and respiratory impacts, elevated temperatures also affect the gastrointestinal (GI) tract, one of the most environmentally sensitive organs (10, 11). Epidemiological studies suggest that heat exposure (HE) impairs digestive and absorptive functions and increases the risk of hospitalization for GI disorders (12, 13). Animal experiments further support this, showing that heat stress disrupts intestinal morphology and induces inflammation and necroptosis (14, 15). The gut microbiota, a complex microbial community within the GI tract, plays a vital role in maintaining intestinal homeostasis and protecting against environmental insults (16, 17). Emerging evidence suggests its involvement in heat-induced intestinal injury. For example, Zang et al. reported that heat stress causes microbial dysbiosis and intestinal inflammation (18), while Wang et al. showed that HE promotes pathogenic bacterial overgrowth and compromises intestinal barrier integrity (19). Moreover, administration of *Lactobacillus rhamnosus* was shown to ameliorate heat-induced intestinal dysfunction in broilers (20). Collectively, these findings suggest that modulating the gut microbiota may be a promising approach for mitigating heat-related intestinal injury.

Fecal microbiota transplantation (FMT), which involves transferring fecal material from healthy donors to recipients, has emerged as an effective intervention to restore microbial balance, thereby enhancing barrier function, regulating immune responses, and reducing intestinal inflammation (21–23). Clinically, it is best known for treating recurrent *Clostridioides difficile* infections (24). However, FMT is not without limitations, including transient gastrointestinal symptoms and rare instances of pathogen transmission (25, 26). Importantly, whether FMT can alleviate heat-induced colonic injury remains unknown.

In this study, we established a heat-exposed mouse model to evaluate whether FMT can attenuate heat-induced colonic injury. Our aim was to investigate the role of gut microbiota in mediating intestinal protection and to explore microbiota-targeted strategies for preventing or treating heat-related gastrointestinal damage.

## MATERIALS AND METHODS

### Experimental animals

Eight-week-old, specific-pathogen-free (SPF) male C57BL/6J mice were purchased from Beijing Vital River Laboratory Animal Technology Co., Ltd. (Beijing, China). The mice were housed in an SPF, climate-controlled facility at Zhejiang Chinese Medical University under a 12-hour light/dark cycle.

### Animal grouping and intervention administration

Mice were randomly assigned to four groups: normal control (NC, 22°C only), normal-FMT (NF, 22°C + FMT), heat exposure (39°C only), and heat exposure-FMT (HF, 39°C + FMT). Each group comprised mice housed in separate cages, with three mice per cage. Mice in the HE and HF groups were exposed to 39°C for 2 hours daily over 15 consecutive days in a temperature-controlled chamber (Jukang, China), while the NC and NF groups underwent the same exposure protocol at 22°C. Outside of these periods, all mice were housed under standard conditions at 22°C.

**Fig 1:**
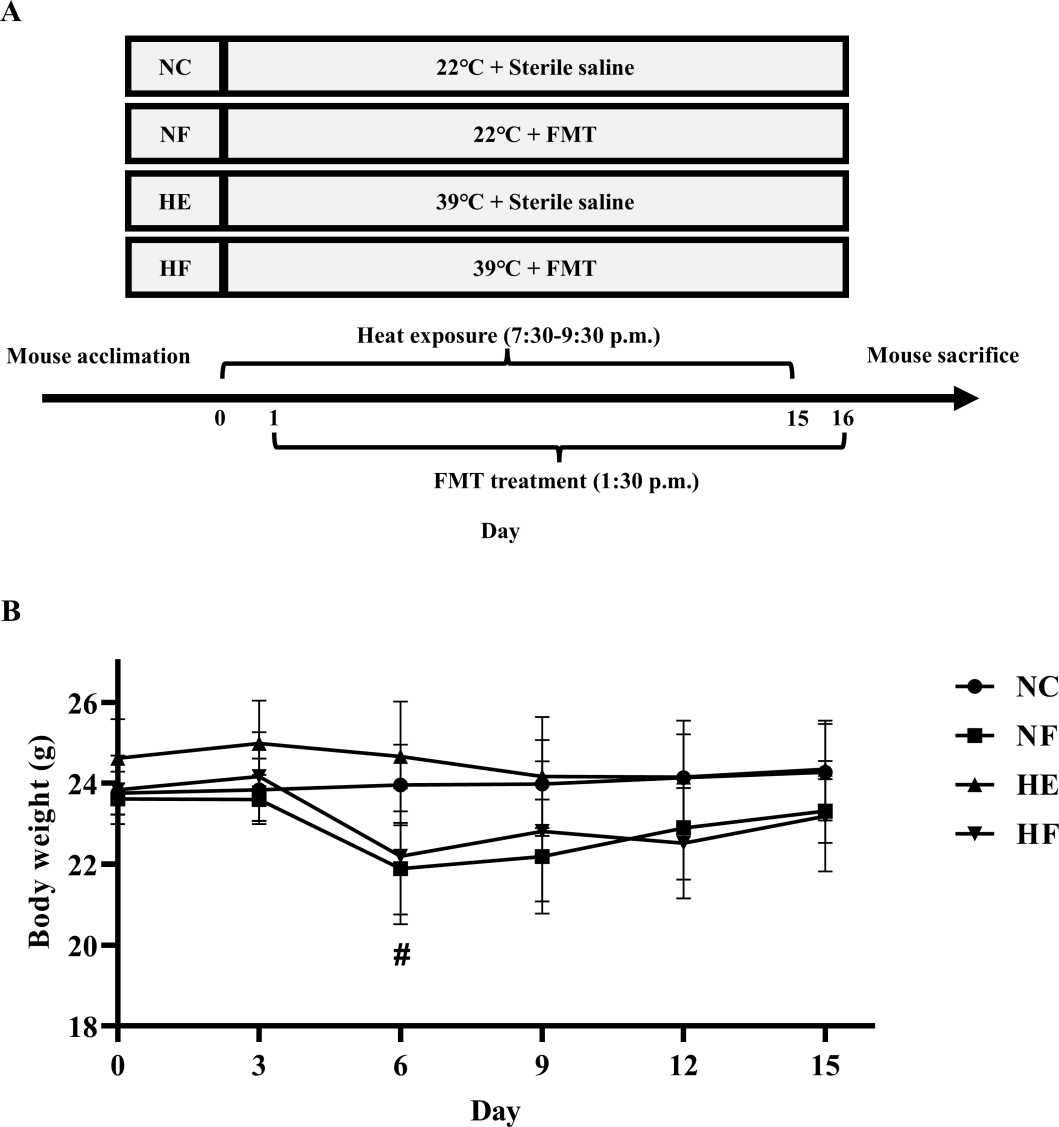
Schematic of experimental design and body weight measurement. (**A**) Schematic of experimental design. (**B**) Mouse body weight measurement. Groups: normal control (22°C only, *n* = 7); NF, 22°C + FMT (*n* = 6); HE (39°C only, *n* = 9); HF, 39°C + FMT (n = 6). Statistical analysis was performed using two-way ANOVA. ^#^*P* < 0.05, NF vs NC.

FMT administration began on the day after the first HE and continued once daily for 15 consecutive days. Mice in the NF and HF groups received 100 µL of freshly prepared FMT suspension via oral gavage, whereas NC and HE groups received an equivalent volume of sterile saline (Beyotime, China). A schematic of experimental design is shown in Fig. 1A.

Fresh fecal pellets were collected from all mice before intervention and pooled after a 1-week acclimation period. The pooled feces were homogenized in sterile saline (100 mg/mL), centrifuged at 1,000 × *g* for 3 min, and the supernatant was filtered through a 70 µm strainer and stored at −80°C. The genus-level composition of the FMT suspension is shown in Fig. S1.

### Sample collection

Body weight was recorded every 3 days throughout the study. Fecal samples were collected aseptically on the final day of the FMT intervention and stored at −80°C for 16S rRNA gene sequencing. At the end of the treatment period, mice were sacrificed. Whole blood was collected, and serum was obtained by centrifugation at 3,000 rpm for 20 min at room temperature, then stored at −80°C. A 1 cm segment of the proximal colon was fixed in 4% paraformaldehyde for histological analysis. The remaining colonic tissue was rinsed with PBS, snap frozen in liquid nitrogen, and stored at −80°C for enzyme-linked immunosorbent assay (ELISA), qPCR, and western blot analyses.

### Biochemical measurements

Serum levels of triglycerides (TG), total cholesterol (TC), fasting blood glucose, alkaline phosphatase (ALP), albumin (ALB), and total protein (TP) were measured using an automated biochemical analyzer (Hitachi, Japan). Colonic secretory immunoglobulin A (sIgA) levels were quantified using ELISA kits (Cusabio, USA) according to the manufacturer’s instructions. Each experimental group included six biological replicates for serum biochemical analyses.

### Histological analysis

Colonic tissue samples were fixed in 4% paraformaldehyde for 48 hours, then dehydrated, embedded in paraffin, and sectioned at 4 µm thickness. Sections were stained with hematoxylin-eosin (H&E) and periodic acid-Schiff (PAS), with five samples analyzed per group. For each mouse, three non-serial sections were prepared, and the section exhibiting the best-preserved morphology, characterized by intact epithelium and continuous mucosal architecture, was selected for analysis. Five randomly chosen fields per section were imaged at ×200 magnification using an optical microscope (Leica Microsystems, Germany).

Tissue injury was scored across three categories: inflammatory cell infiltration, goblet cell count, and mucosal architecture, according to a modified scoring system adapted from previous studies (27–29) detailed in Table S1. Inflammatory infiltration and mucosal structure were evaluated on H&E-stained sections, while goblet cells were quantified in PAS-stained crypts. All scoring was conducted in a blinded manner by two independent researchers.

### Terminal deoxynucleotidyl transferase deoxyuridine triphosphate nick end labeling staining

Apoptotic cells in colonic tissue were detected using a transferase deoxyuridine triphosphate nick end labeling (TUNEL) apoptosis detection kit (Vazyme, China). After antigen retrieval, tissue sections were incubated overnight at 4°C in the dark with TdT incubation buffer. Subsequently, nuclei were counterstained with 4′,6-diamidino-2-phenylindole (DAPI) for visualization. Slides were mounted with anti-fade medium. Apoptotic cells, indicated by green fluorescence, were observed under a fluorescence microscope (Leica Microsystems, Germany) at ×200 magnification. For each sample, one representative section was analyzed by selecting five random fields. Quantification was performed in a blinded manner.

### Immunohistochemical staining

Immunohistochemical (IHC) staining was performed to detect 3-nitrotyrosine (3-NT) expression in colon tissue sections. After deparaffinization and antigen retrieval, sections were incubated overnight at 4°C with anti-3-NT antibody (1:100, Abcam, Cat# ab110282), followed by incubation with a horseradish peroxidase (HRP)-conjugated secondary antibody. Signals were developed using 3,3′-diaminobenzidine as the chromogenic substrate. Images were captured at ×200 magnification using an optical microscope. Quantification of the stained area and integrated optical density (IOD) was performed using ImageJ software (NIH, USA). The average densitometric value (IOD/area) was calculated to represent 3-NT expression intensity. Analysis was conducted in a blinded manner on five randomly selected fields per section.

### RNA isolation and qRT-PCR

Total RNA was extracted from colonic tissues using RNAiso Plus reagent (Takara Bio Inc., Japan) according to the manufacturer’s instructions. Complementary DNA was synthesized from 500 ng of total RNA using PrimeScript RT Master Mix (Takara Bio Inc.). Quantitative real-time PCR was performed on a QuantStudio 7 Flex system (Applied Biosystems, USA) using SYBR Green detection. Relative gene expression levels were calculated using the 2^−ΔΔCt^ method with β-actin as the internal reference gene. Primer sequences are listed in Table S2. Each experimental group included six biological replicates.

### Western blot

Colon tissues were homogenized in radio immunoprecipitation assay lysis buffer (Bosterbio, USA), and total protein concentrations were determined using the BCA Protein Assay Kit (Beyotime, China). Equal amounts of protein samples were separated by 10% SDS-PAGE and transferred onto polyvinylidene difluoride membranes. Membranes were blocked and incubated overnight at 4°C with primary antibodies diluted in Tris-buffered saline with Tween-20 (TBST). The following primary antibodies were used: Caspase-3 (1:2,000, Proteintech, Cat# 19677-1-AP), Caspase-9 (1:1,000, Proteintech, Cat# 10380-1-AP), Bcl-2 (1:4,000, Proteintech, Cat# 68103-1-Ig), Bak (1:8,000, Proteintech, Cat# 29552-1-AP), Bax (1:8,000, Proteintech, Cat# 50599-2-Ig), phospho-P53 (p-P53; 1:2,000, CST, Cat# 9284S), total P53 (1:2,000, CST, Cat# 2524T), and β-actin (1:20,000, Abcam, Cat# ab8227). After incubation with appropriate HRP-conjugated secondary antibodies, signals were detected using a chemiluminescence imaging system (Bio-Rad, USA). Band intensities were quantified using ImageJ software and normalized to β-actin. All experiments were performed with at least six independent biological replicates per group.

### Fecal DNA extraction and 16S rRNA sequencing

Microbial genomic DNA was extracted from freshly collected fecal samples using a commercial DNA extraction kit (Magen, China) according to the manufacturer’s instructions. The quantity and purity of the extracted DNA were assessed using a NanoDrop 2000 spectrophotometer (Thermo Fisher Scientific, USA) and verified by 1% agarose gel electrophoresis. The V3-V4 hypervariable regions of the bacterial 16S rRNA gene were amplified using region-specific primers 343F (5′-TACGGRAGGCAGCAG-3′) and 798R (5′-AGGGTATCTAATCCT-3′) with unique barcodes. The purified amplicons were pooled in equimolar concentrations and subjected to paired-end sequencing on the Illumina NovaSeq 6000 platform (Illumina, USA).

### Bioinformatics analysis

Raw reads were processed using QIIME2 (v1.9.1). Primer sequences were trimmed using the q2-cutadapt plugin. Denoising, merging, and chimera removal were performed using the DADA2 pipeline. Taxonomic classification was conducted via the q2-feature-classifier classify-sklearn method, with a Naive Bayes classifier trained on the SILVA database (release 138, https://www.arb-silva.de/), using a 100% sequence identity threshold. Default parameters were applied unless otherwise stated.

Analyses were performed based on the relative abundance of amplicon sequence variants. To adjust for batch effects, the ConQuR framework was employed (30). α-Diversity was assessed using the Chao1 index and compared by two-way ANOVA. β-Diversity was evaluated via principal coordinates analysis based on the binary Jaccard index, with statistical significance determined using the Adonis test. Taxonomic compositions at the phylum and genus levels were visualized through stacked bar plots. Differentially abundant genera were identified using MaAsLin2, which accounts for fixed and random effects while controlling for multiple testing using the Benjamini-Hochberg procedure; taxa with *q*-values < 0.25 were considered significant. Genera exhibiting significant main or interaction effects (HE, FMT, or HE × FMT) were visualized using heatmaps. Follow-up pairwise comparisons between the NC and HE groups, and between the HE and HF groups, were conducted using Fisher’s least significant difference test and visualized with bar plots.

### Statistical analysis

All general statistical analyses and data visualizations were performed using GraphPad Prism 9 (San Diego, CA, USA). Data are presented as mean ± SD, unless otherwise stated. Two-way ANOVA was used to evaluate the main effects of HE, FMT, and their interaction on biochemical and molecular data. Spearman’s correlation analysis was conducted to assess associations between gut microbiota and oxidative stress markers, with correlation matrices visualized using the *pheatmap* package in R. Correlations were considered statistically significant when Spearman’s *ρ* > 0.80 or < –0.80 and *P* < 0.05.

## RESULTS

### Body weight and circulating biochemical analysis

As shown in Fig. 1B, FMT intervention caused a transient reduction in body weight during the early treatment period; however, body weight gradually returned to baseline levels by the end of the experiment. Serum biochemical parameters, including TG, TC, and ALP, showed no significant differences among groups. Notably, HE significantly decreased fasting glucose and TP levels, while FMT treatment reduced ALB levels (Fig. S2).

### Microbiota profile

**Fig 2:**
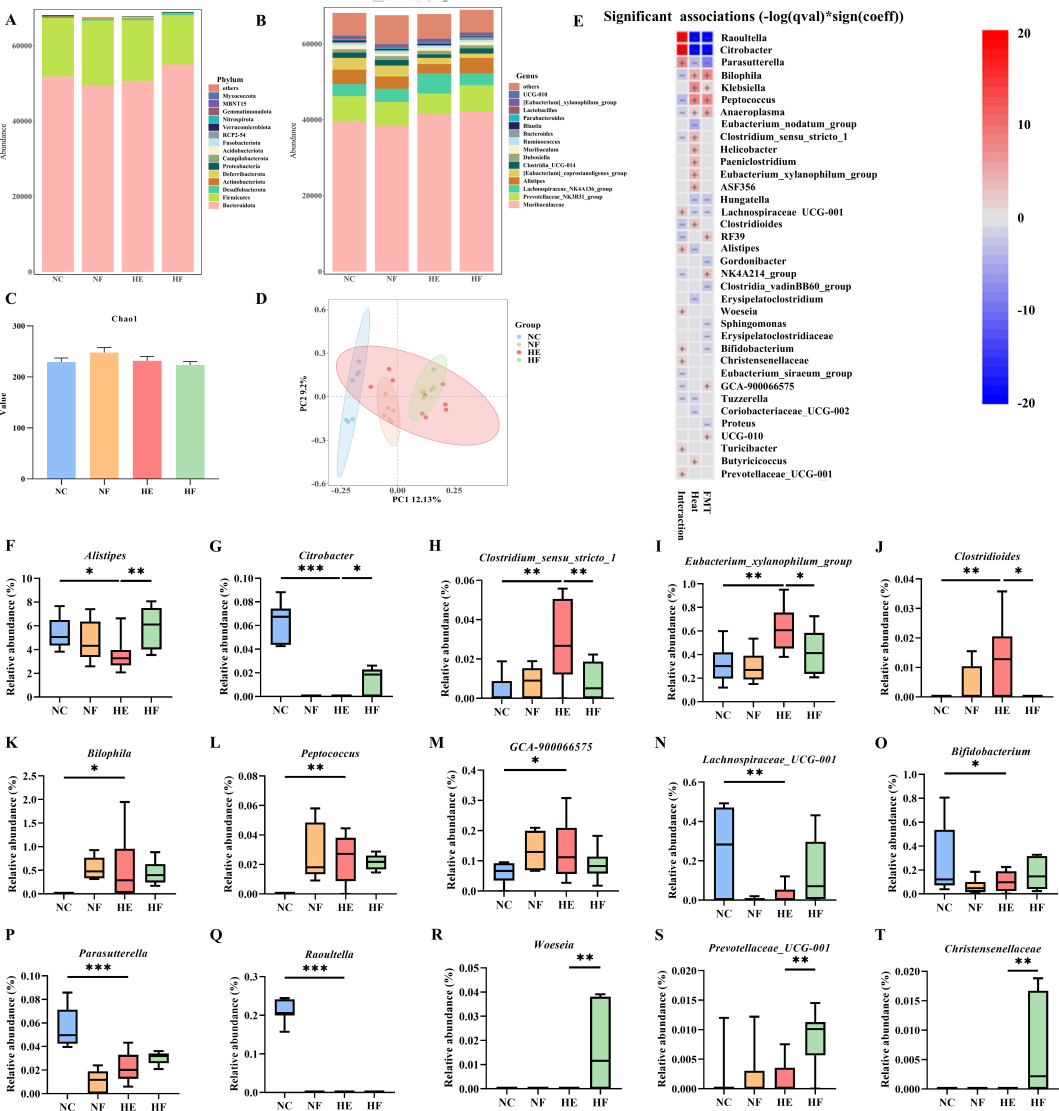
FMT modulated gut microbiota dysbiosis in mice induced by HE. (**A**) α-Diversity analysis of gut microbiota using Chao1 index. (**B**) β-Diversity analysis via binary Jaccard Adonis. (**C**) Relative abundances of gut microbiota at the phylum level across groups. (**D**) Relative abundances of gut microbiota at the genus level across groups. (**E**) Heatmap of genus-level taxa significantly associated with HE, FMT, or their interaction using MaAsLin2 analysis. Red indicates a positive association, while blue indicates a negative association. (**F–T**). Relative abundance of representative genera *Alistipes* (**F**), *Eubacterium_xylanophilum_group* (**G**), *Citrobacter* (**H**), *Clostridium_sensu_stricto_1* (**I**), *Clostridioides* (**J**), *Bilophila* (**K**), *Raoultella* (**L**), *Parasutterella* (**M**), *Peptococcus* (**N**), *Bifidobacterium* (**O**), *GCA-900066575* (**P**), *Lachnospiraceae_UCG-001* (**Q**), *Woeseia* (**R**), *Prevotellaceae_UCG-001* (**S**), and *Christensenellaceae* (**T**). Groups: normal control (22°C only, *n* = 7); NF, 22°C + FMT (*n* = 6); HE (39°C only, *n* = 9); HF, 39°C + FMT (*n* = 6). Statistical analysis was performed using two-way ANOVA and MaAsLin2. *P < 0.05, **P < 0.01, and ***P < 0.001.

To evaluate microbial changes induced by HE and FMT, 16S rRNA gene sequencing was performed. At the phylum level, Bacteroidota, Firmicutes, and Desulfobacterota were the predominant fecal microbiota (Fig. 2A). At the genus level, the most abundant taxa included *Muribaculaceae, Prevotellaceae_NK3B31*, *Lachnospiraceae_NK4A136*, *Alistipes*, and *[Eubacterium]_coprostanoligenes_group* (Fig. 2B). α-Diversity analysis revealed no significant differences in microbial richness or diversity among the groups (Fig. 2C), whereas β-diversity analysis demonstrated significant compositional differences, as confirmed by the Adonis test (*R*² =0.237, *P* = 0.001) (Fig. 2D).

MaAsLin2 analysis identified several genera significantly associated with the main effects of HE, FMT, or their interaction (*q* < 0.25). Genera such as *Alistipes*, *Bifidobacterium*, and *Lachnospiraceae_UCG-001* exhibited positive interaction coefficients, indicating that their abundance increased specifically in the FMT-treated group under HE. Conversely, *Bilophila*, *Peptococcus*, and *Clostridium_sensu_stricto_1* showed negative interaction coefficients, indicating that FMT reduced their abundance under heat stress (Fig. 2E; Table S3).

Pairwise comparisons revealed that HE significantly decreased the relative abundance of *Alistipes* and *Citrobacter* while increasing *Clostridium_sensu_stricto_1*, *Eubacterium_xylanophilum_group,* and *Clostridioides* (*P* < 0.05); these changes were substantially reversed by FMT (*P* < 0.05; *q* for interaction <0.05; Fig. 2F through J). HE also significantly increased *Bilophila, Peptococcus,* and *GCA-900066575* and decreased *Lachnospiraceae_UCG-001*, *Parasutterella, Bifidobacterium,* and *Raoultella* (*P* < 0.05); although pairwise comparisons between HF and HE for these taxa did not reach significance (*P* > 0.05; Fig. 2K through Q), reversal trends after FMT were supported by significant interaction effects (*q* for interaction < 0.05). Additionally, FMT significantly increased the relative abundance of *Woeseia*, *Christensenellaceae*, and *Prevotellaceae_UCG-001* (*P* < 0.05), although these genera were not significantly affected by HE alone (*P* > 0.05; Fig. 2R through T).

### FMT alleviated colonic tissue damage induced by HE

**Fig 3:**
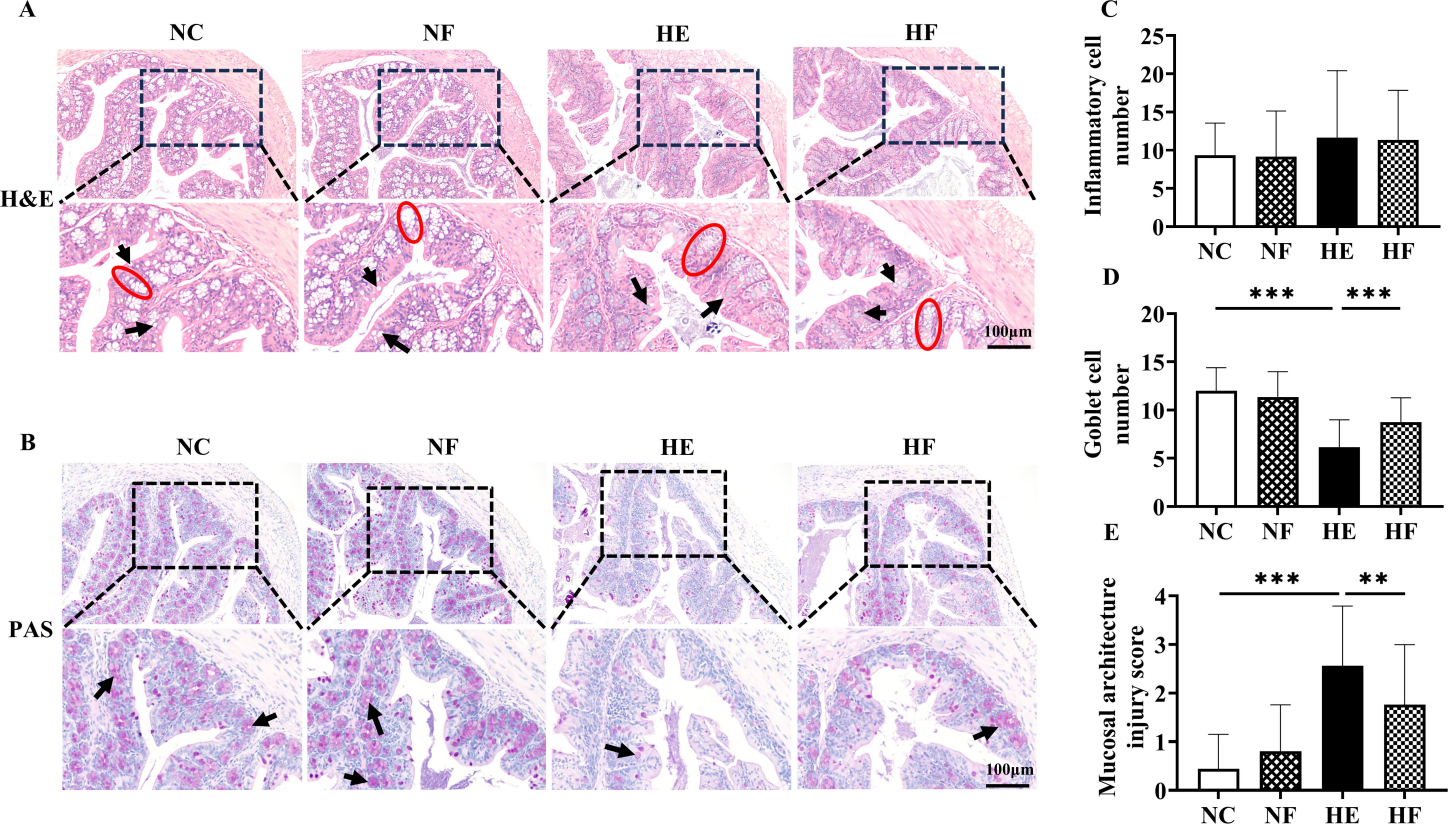
FMT alleviated colonic tissue damage induced by HE. (**A**) Representative images of hematoxylin-eosin staining of colonic tissue sections (×100, insert ×200). Black arrows point to goblet cells, and red ovals point to crypts. (**B**) Representative images of periodic acid-Schiff staining of colonic tissue sections (×100, insert ×200). Black arrows point to goblet cells. (**C**) Inflammatory cell number. (**D**) Goblet cell number. (**E**) Mucosal architecture injury score. Groups: normal control (22°C only); NF, 22°C + FMT; HE (39°C only); HF, 39°C + FMT; *n* = 5. Statistical analysis was performed using two-way ANOVA. **P < 0.01 and ***P < 0.001.

Histological analysis of colonic tissues using H&E and PAS staining revealed that, in the HE group, the colonic mucosal surface exhibited irregular villous or cuboidal morphology, and crypts displayed disorganized structures with widened openings and disrupted architecture. In contrast, FMT markedly restored mucosal integrity and crypt organization (Fig. 3A, B, and E). The number of goblet cells and mucus secretion was significantly reduced in the HE group compared to the NC group but was notably improved following FMT intervention (Fig. 3D). Minimal inflammatory cell infiltration was observed across all groups (Fig. 3C). Although HE decreased the expression of tight junction proteins (*Claudin-1*, *Zo-1*), secretory IgA (sIgA), and *Mucin 1*, these reductions were not significantly reversed by FMT treatment (Fig. S3).

### FMT alleviated colonic epithelial cell apoptosis induced by HE

**Fig 4:**
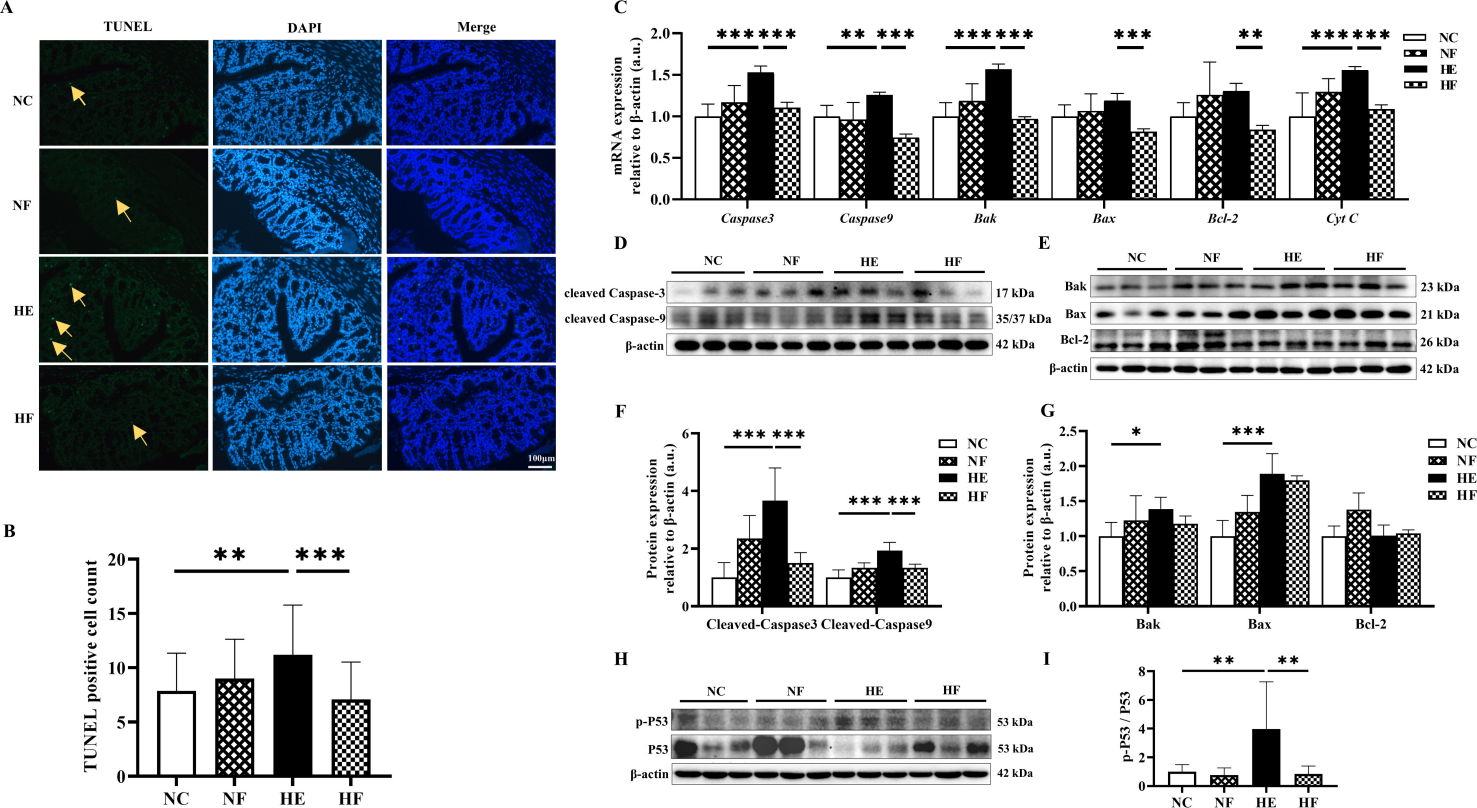
FMT reduced colonic epithelial cell apoptosis induced by HE. (**A**) Representative images of terminal deoxynucleotidyl transferase dUTP nick end labeling staining in colonic tissue sections (×200). Yellow arrows point to apoptosis cells. (**B**) TUNEL positive cell count. (**C**) mRNA levels of *Caspase-3*, *Caspase-9*, *Bak*, *Bax*, *Bcl-2*, and *Cyt C*. (**D and E**) Representative bands (**D**) and quantitative analysis of cleaved Caspase-3 and cleaved Caspase-9 (**E**). (**F and G**) Representative bands (**F**) and quantitative analysis of Bak, Bax, and Bcl-2 (**G**). (**H and I**) Representative bands (**H**) and quantitative analysis of p-P53 normalized to P53 (**I**). Groups: normal control (22°C only); NF, 22°C + FMT; heat exposure (39°C only); HF, 39°C + FMT; *n* = 5 (**B**), *n* = 6 (**C–E, H**). Statistical analysis was performed using two-way ANOVA. *P < 0.05, **P < 0.01, and ***P < 0.001.

To further investigate the protective effects of FMT on colonic epithelial cell apoptosis, TUNEL staining was performed. A significant increase in apoptotic cells was observed in the colonic mucosa of the HE group, which was markedly reduced following FMT intervention (Fig. 4A and B). Consistently, HE significantly upregulated the mRNA expression of pro-apoptotic markers, including *Caspase-3*, *Caspase-9*, *Bak*, and *Cytochrome c* (*Cyt C*), while these changes were effectively reversed by FMT treatment (Fig. 4C).

At the protein level, cleaved caspase-3, cleaved caspase-9, Bak, Bax, and the p-P53/P53 ratio were significantly elevated in the HE group. FMT intervention significantly suppressed the increases in cleaved Caspase-3, cleaved Caspase-9, and p-P53/P53 ratio (Fig. 4D through I). Although reductions in Bak and Bax following FMT did not reach statistical significance, a downward trend was observed with significant interaction effects (*P* for interaction < 0.05). The complete protein blots for all samples are provided in Fig. S4.

### FMT inhibited oxidative stress induced by HE

**Fig 5:**
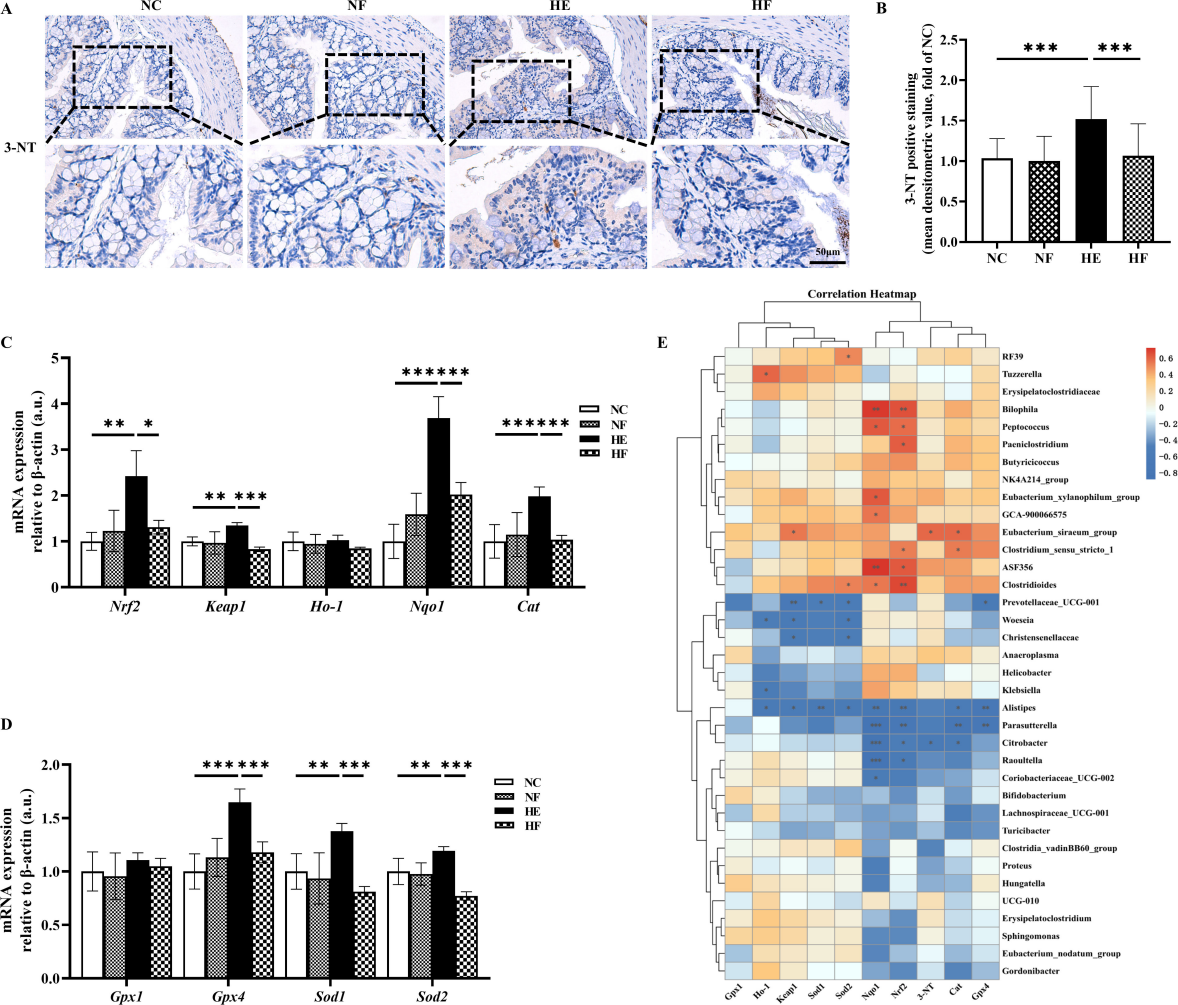
FMT inhibited oxidative stress induced by HE. (**A**) Representative images of immunohistochemistry staining for 3-nitrotyrosine in colonic tissue sections (×200, insert ×400). (**B**) The analysis of IHC staining intensity of 3-NT. (**C**) mRNA levels of *Nrf2*, *Keap1*, *Ho-1*, *Nqo1*, and *Cat*. (**D**) mRNA levels of *Gpx1*, *Gpx4*, *Sod1*, and *Sod2*. (**E**) Heatmap showing Spearman’s correlation between differential microbiota abundance at genus level and oxidative stress. The coefficient (*ρ*) is displayed in different colors in the figure. *ρ* >0.8 or *ρ* <−0.8, and *P* < 0.05 were considered as a significant correlation. Groups: normal control (22°C only); NF, 22°C + FMT; heat exposure (39°C only); HF, 39°C + FMT; *n* = 5 (**B**), *n* = 6 (**C and D**). Statistical analysis was performed using two-way ANOVA. *P < 0.05, **P < 0.01, and ***P < 0.001.

IHC analysis of 3-NT revealed strong staining in the colonic mucosa of heat-exposed mice, indicating elevated oxidative stress. Importantly, FMT treatment significantly alleviated this oxidative stress, demonstrating its protective effect (Fig. 5A and B). Furthermore, HE significantly upregulated the mRNA expression of the transcription factor nuclear factor erythroid 2-related factor 2 (*Nrf2*), its upstream regulator kelch-like ECH-associated protein 1 (*Keap1*), and several downstream antioxidant enzymes, including NAD(P)H quinone dehydrogenase 1 (*Nqo1*), catalase (*Cat*), glutathione peroxidase 4 (*Gpx4*), superoxide dismutase 1 (*Sod1*), and superoxide dismutase 2 (*Sod2)*. FMT intervention effectively restored the expression levels of these genes to near-baseline levels (*P* < 0.05; Fig. 5C and D).

The differential microbial genera identified by MaAsLin2 were further examined by Spearman’s correlation analysis against 3-NT, *Nrf2*, and downstream antioxidant genes. The resulting heatmap revealed distinct associations between specific taxa and oxidative stress markers (Fig. 5E). Notably, *Alistipes* showed strong negative correlations with nearly all oxidative stress-related markers. *Citrobacter* and *Parasutterella* were negatively correlated with *Nqo1*, *Nrf2*, and *Cat*, while *Prevotellaceae_UCG-001*, *Christensenellaceae,* and *Woeseia* showed inverse correlations with *Keap1* and *Sod2*. Conversely, *Bilophila*, *Peptococcus,* and *Clostridioides* displayed significant positive correlations with *Nqo1* and *Nrf2*. Additionally, *Clostridium_sensu_stricto_1* correlated positively with *Cat*, and *Eubacterium_xylanophilum_group* showed positive correlation with *Nqo1*.

## DISCUSSION

In this study, we demonstrated that FMT effectively alleviated colonic injury induced by HE in C57BL/6J mice. Specifically, FMT intervention reshaped the gut microbial composition, restored colonic mucosal integrity, attenuated epithelial cell apoptosis, and mitigated oxidative stress induced by HE. These findings underscore the therapeutic potential of FMT in preserving intestinal homeostasis and protecting the gut from heat stress-associated damage.

Previous studies have highlighted the clinical benefits of FMT in promoting gut health (31, 32). In addition to its well-established efficacy against recurrent *Clostridioides difficile* infection, FMT has shown therapeutic potential in various gastrointestinal conditions, including inflammatory bowel disease, chronic constipation, and short bowel syndrome, as well as metabolic disorders such as dyslipidemia and insulin resistance. The underlying mechanisms may include restoration of microbial diversity, competitive inhibition of pathogenic bacteria, modulation of host metabolic functions, and rebalancing of immune responses (24, 33, 34). Several studies suggest that FMT may offer therapeutic efficacy beyond those of conventional pharmacological treatments (35, 36). However, the role of FMT in heat-induced intestinal injury remains largely unexplored. Our study addresses this gap by providing direct evidence that FMT mitigates HE-induced colonic injury in mice.

We identified several bacterial taxa that are associated with FMT that may contribute to the alleviation of HE-induced intestinal injury. Among these, *Alistipes* showed the highest relative abundance. *Alistipes* is increasingly recognized for its role in maintaining gut barrier integrity and suppressing intestinal inflammation (37–39). Consistent with this, our data showed that *Alistipes* abundance was markedly reduced after HE but was significantly restored following FMT, suggesting its potential involvement in mitigating colonic injury and contributing to the protective effects of FMT. Similarly, *Bifidobacterium* and *Lachnospiraceae_UCG-001*, both known short-chain fatty acid (SCFA)-producing genera, were significantly depleted in the HE group but increased following FMT. SCFAs such as butyrate are crucial for colonocyte energy supply, tight junction protein expression, and anti-apoptotic signaling. The enrichment of these genera may therefore facilitate epithelial regeneration and enhance intestinal barrier restoration (40, 41). In contrast, *Bilophila*, a genus frequently associated with gut inflammation and metabolic endotoxemia, particularly under high-fat diets, was elevated after HE but substantially decreased following FMT. This suggests that FMT may suppress the expansion of pro-inflammatory bacteria, thereby rebalancing the microbial ecosystem toward a more homeostatic and anti-inflammatory state (42, 43). In addition, several low-abundance taxa (relative abundance < 0.1%), including *Citrobacter*, *Prevotellaceae_UCG-001, and Clostridium_sensu_stricto_1,* were significantly modulated by FMT. Although these taxa are minor in relative abundance, emerging evidence suggests they may play disproportionate roles in host physiology, including modulation of oxidative stress, immune signaling, and microbial metabolic networks (44, 45). The changes observed in these taxa following FMT may reflect the involvement of complex and multilayered microbial mechanisms in the protective effects of FMT against heat-induced colonic injury.

The intestinal mucosal epithelium constitutes a critical first line of defense against luminal pathogens, toxins, and mechanical insults, with goblet cells in the crypts secreting mucus to form a protective barrier. Consistent with previous studies, our results show that HE impairs the mucosal barrier and reduces goblet cell numbers and its function (46–50). Importantly, FMT effectively mitigates these adverse effects by preserving mucosal integrity and restoring goblet cell abundance and activity. These protective effects may be mediated through direct microbial stimulation of mucus secretion or modulation of key microbial metabolites, such as SCFAs. However, we did not observe a complete restoration of tight junction proteins (e.g., Claudin-1, ZO-1, and Muc1) in our model after FMT, indicating that FMT may only partially reverse heat-induced mucosal barrier impairment.

While apoptosis is a normal physiological process involved in epithelial renewal, excessive apoptosis can cause epithelial erosion and compromise mucosal integrity (51). Notably, FMT significantly reduced HE-induced epithelial apoptosis, as evidenced by the downregulation of pro-apoptotic markers such as caspase-3 and caspase-9, underscoring its protective role in maintaining mucosal homeostasis. These findings align with previous reports in other models. For instance, Chen et al. found that FMT inhibited neuronal apoptosis in the rats with ischemic stroke (52). Li et al. showed that FMT suppressed intestinal epithelial apoptosis and bacterial translocation, thereby ameliorating necrotizing enterocolitis (53).

Oxidative stress refers to an imbalance between reactive oxygen species (ROS) production and antioxidant defenses, leading to cellular damage, including lipid peroxidation, protein oxidation, and DNA damage (54). Under heat stress, excessive ROS generation overwhelms the antioxidant defenses capacity, causing epithelial cell apoptosis and mucosal barrier dysfunction in the gut (55–57). Key oxidative stress markers include 3-NT, Nrf2, and downstream antioxidant enzymes, such as SOD, NQO1, HO-1, and GPx. In our study, FMT significantly normalized the expression of these markers, contributing to the restoration of epithelial homeostasis and resilience against oxidative damage caused by HE.

Many studies have shown that gut microbiota can influence host oxidative stress. For example, some bacteria have been reported to induce antioxidant enzymes, such as SOD and GPx, while others may be linked to increased ROS production through NADPH oxidase activation triggered by formylated peptides (58–60). Consistent with these findings, our study found that genera such as *Alistipes* and *Parasutterella* were negatively correlated with oxidative stress markers (3-NT, Nrf2, and SODs), suggesting a protective role against HE-induced redox imbalance. In contrast, *Bilophila, Peptococcus,* and *Clostridioides* showed positive correlations, indicating potential pro-oxidative effects.

Notably, mice receiving FMT showed a more pronounced and rapid decline in body weight compared to the non-FMT group. This change may partly result from microbiota-mediated alterations in host energy metabolism and feeding behavior. However, procedural factors related to FMT administration, such as gavage-induced stress, the physical characteristics of the suspension, or microbial load, could also contribute to transient weight loss. Reduced body weight may decrease adiposity, potentially enhancing heat dissipation and attenuating systemic metabolic and inflammatory responses, thereby improving resilience to heat stress. Thus, while gut microbiota modulation likely plays a key role in FMT’s protective effects on colonic injury, these additional factors should also be taken into account (61, 62).

This study has several strengths. First, it systematically demonstrates the protective role of FMT against heat-induced colonic injury by integrating gut microbiota profiling with assessments of epithelial apoptosis, mucosal integrity, and oxidative stress. Second, it identifies specific gut bacterial taxa associated with attenuation of heat-induced damage, providing mechanistic insights that enhance our understanding of microbiota-mediated intestinal protection.

However, several limitations of this study should be acknowledged. First, each mouse was treated as an individual sample rather than using the cage as the experimental unit, which may introduce potential cage effects. Mice housed together can share microbes and develop similar microbiota profiles, potentially reducing the effective sample size and affecting statistical power (63, 64). In addition, while using a pooled FMT inoculum from multiple donors facilitates standardization, it may limit the generalizability of our findings. Future studies employing randomized cage allocation and single-donor FMTs could improve reproducibility and translatability. Second, the absence of selective bacterial transplantation and longitudinal microbiota profiling restricts mechanistic insights into specific taxa and microbial dynamics. Targeted microbial validation will be essential to clarify the causal roles of key commensals. Finally, the precise molecular mechanisms underlying FMT-mediated epithelial protection remain incompletely understood. Integration of multi-omics approaches combined with host signaling analyses is warranted to elucidate these complex host-microbe interactions.

### Conclusion

This study is the first to demonstrate the protective effects of FMT against heat-induced colonic injury. These results highlight the critical role of gut microbiota in heat-induced colonic damage and offer new insights into potential therapeutic strategies.

## Data Availability

Raw sequence reads have been deposited in the BioProject database with ID PRJNA1226403.

## SUPPLEMENTAL MATERIAL

Supplemental material aem.00976-25-s0001.docx Tables S1 to S3; Figures S1 to S4.

ASM does not own the copyrights to Supplemental Material that may be linked to, or accessed through, an article. The authors have granted ASM a non-exclusive, world-wide license to publish the Supplemental Material files. Please contact the corresponding author directly for reuse.
