# Supplementary material for: Fecal microbiota transplantation alleviated heat-induced colonic tissue damage, epithelial apoptosis, and oxidative stress
Source: Appl Environ Microbiol. 2025 Sep 24;91(10):e00976-25. doi: 10.1128/aem.00976-25 (PMC12542630; doi:10.1128/aem.00976-25)
Supplement: Supplemental material — Tables S1 to S3; Figures S1 to S4. [file aem.00976-25-s0001.docx]

| Category | Criterion | Description | Score value |
| --- | --- | --- | --- |
| Inflammatory cell infiltration | Number | Leukocyte number of lamina propria area infiltrated |  |
|  | Extent | Expansion of leukocyte infiltration: |  |
|  |  | No infiltration | 0 |
|  |  | Mucosal | 1 |
|  |  | Mucosal and submucosal | 2 |
|  |  | Mucosal, submucosal and transmural | 3 |
| Goblet cells | Number | Goblet cell number from well-oriented samples with longitudinally cut crypts only^1^. |  |
| Mucosal architecture | Mucosal epithelium changes | Intactness of mucosal epithelium: |  |
|  |  | Intact, columnar epithelium | 0 |
|  |  | Uneven, villous or finger-like changes | 1 |
|  |  | Loss of surface epithelium | 2 |
|  | Crypt changes | Structural changes and arrangement of crypts: |  |
|  |  | Straight, parallel and extending seamlessly from just above the muscularis mucosae to the surface | 0 |
|  |  | Irregularly arranged crypts | 1 |
|  |  | Crypt branching^2^, crypt distortion^3^, crypt atrophy^4^ | 2 |
|  |  | Mucosa devoid of crypts | 3 |

Table S1. Histopathological scores of colonic injury evaluation in mice

^1^Five comparable crypts per field were analyzed. ^2^More than two branched crypts in a well-oriented biopsy specimen with a muscularis mucosae length of at least 2 mm. ^3^Non-parallel, variable diameter, or cystically dilated crypts, contrasting with normal appearance. ^4^Increased, typically variable distance between crypt bases and the muscularis mucosae.

Table S2. Primer sequence for qRT-PCR

| Target gene | Forward primer | Reverse primer |
| --- | --- | --- |
| *β-actin* | TGTGATGGTGGGAATGGGTCAGAA | TGTGGTGCCAGATCTTCTCCATGT |
| *Zo-1* | GATGTTTATGCGGACGGTGG | CATTGCTGTGCTCTTAGCGG |
| *Claudin1* | TGAAGTGCATGAGGTGCCTG | CACTAATGTCGCCAGACCTGAAA |
| *Occludin* | CCCCTCTTTCCTTAGGCGACA | CTCCCAAGATAAGCGAACCTGC |
| *Muc1* | CCTACCTACCACACTCACGG  ACCTGGAAGGCCCAATCAAG | ATTACCTGCCGAAACCTCCTC |
| *Muc2* |  | CTCAGCGTAGTTGGCACTCT |
| *Caspase3* | GCTTGGAACGGTACGCTAAG | CCACTGACTTGCTCCCATGT |
| *Caspase9* | TCCTGGTACATCGAGACCTTG | AAGTCCCTTTCGCAGAAACAG |
| *Bak* | CAAGATCGCCTCCAGCCTATT | CACGCTGGTAGACGTACAGG |
| *Bax* | AGACAGGGGCCTTTTTGCTAC | AATTCGCCGGAGACACTCG |
| *Bcl-2* | GTGTGGAGAGCGTCAACAGG | ATATAGTTCCACAAAGGCATCCCAG |
| *Cyt C* | CCAGCCCGGAACGAATTAAA | CCGTGGAGATTTGGTCCAGT |
| *Nrf2* | TGGGCAACCATCACTCTGCT | TCTGCTGCAAGTAGCCTCG |
| *Keap1* | GGTTCGGTTACCGTCCTGC | TGCCCCTGTGGTCAAAGTG |
| *Ho-1* | AAGCTGAGAGTGAGGACCCA | GCTAGCCTGGTGCAAGATACT |
| *Nqo1* | TGCTAGAGATGACTCGGAAGG | AGGATGGGAGGTACTCGAATC |
| *Cat* | GCCCTGGTCGGTCTTGTAAT | ATGGTCACCGGCACATGAAT |
| *Gpx1* | AGAGAGACGCGACATTCTCAAT | CCACCGTGTATGCCTTCTCC |
| *Gpx4* | ATGCACACGAAACCCCTGTA | GTACTGCAACAGCTCCGAGT |
| *Sod1* | GGTTCACCGCTTGCCTTCTG | GGGAAGCATGGCGATGAAAG |
| *Sod2* | GCCTGAACCTTGGACTCCC | TGGAGAACCCAAAGGAGAGTTG |

Table S3. Relative abundance of taxonomy at genus level

| Taxonomy | Relative abundance (%) | | | |  | *q*-value | | |  |
| --- | --- | --- | --- | --- | --- | --- | --- | --- | --- |
|  | NC | NF | HE | HF |  | Heat | FMT | Interaction | |
| *Alistipes* | 5.532 | 4.711 | 3.544 | 5.885 |  | 0.083* | 0.659 | 0.086* | |
| *Eubacterium_xylanophilum_group* | 0.319 | 0.295 | 0.613 | 0.423 |  | 0.038* | 0.983 | 0.649 | |
| *UCG-010* | 0.444 | 0.570 | 0.381 | 0.313 |  | 0.575 | 0.241* | 0.500 | |
| *Bilophila* | 0.010 | 0.541 | 0.556 | 0.444 |  | 0.007* | < 0.001* | 0.069* | |
| *Eubacterium_siraeum_group* | 0 | 1.028 | 0.479 | 0 |  | 0.515 | 0.341 | 0.224* | |
| *Anaeroplasma* | 0.052 | 0.645 | 0.311 | 0.401 |  | 0.241* | 0.013* | 0.174* | |
| *Clostridia_vadinBB60_group* | 0.374 | 0.150 | 0.304 | 0.266 |  | 0.646 | 0.113* | 0.461 | |
| *Eubacterium_nodatum_group* | 0.345 | 0.264 | 0.199 | 0.186 |  | 0.017* | 0.421 | 0.735 | |
| *Bifidobacterium* | 0.285 | 0.063 | 0.104 | 0.167 |  | 0.360 | 0.241* | 0.222* | |
| *Tuzzerella* | 0.191 | 0.175 | 0.138 | 0.008 |  | 0.225* | 1.000 | 0.241* | |
| *GCA-900066575* | 0.061 | 0.134 | 0.135 | 0.088 |  | 0.264 | 0.241* | 0.224* | |
| *Lachnospiraceae_UCG-001* | 0.234 | 0.004 | 0.025 | 0.139 |  | 0.241* | 0.160* | 0.067* | |
| *NK4A214_group* | 0.062 | 0.124 | 0.080 | 0.091 |  | 0.494 | 0.111* | 0.241* | |
| *Erysipelatoclostridium* | 0.116 | 0.077 | 0.056 | 0.045 |  | 0.123* | 0.735 | 0.932 | |
| *Helicobacter* | 0.027 | 0.034 | 0.076 | 0.088 |  | 0.030* | 0.792 | 1.000 | |
| *Turicibacter* | 0.073 | 0.039 | 0.037 | 0.071 |  | 0.421 | 0.583 | 0.241* | |
| *Raoultella* | 0.211 | 0 | 0 | 0 |  | < 0.001* | < 0.001* | < 0.001* | |
| *Klebsiella* | 0.013 | 0.030 | 0.060 | 0.093 |  | < 0.001* | 0.222* | 0.932 | |
| *ASF356* | 0.012 | 0.017 | 0.055% | 0.042 |  | 0.043* | 0.932 | 0.737 | |
| *Parasutterella* | 0.057 | 0.011 | 0.022 | 0.030 |  | 0.021* | < 0.001* | < 0.001* | |
| *Coriobacteriaceae_UCG-002* | 0.068 | 0.030 | 0.012 | 0.009 |  | 0.225* | 0.735 | 0.925 | |
| *Citrobacter* | 0.065 | 0 | 0 | 0.014 |  | < 0.001* | < 0.001* | < 0.001* | |
| *Peptococcus* | 0 | 0.027 | 0.023 | 0.022 |  | < 0.001* | < 0.001* | 0.030* | |
| *Clostridium_sensu_stricto_1* | 0.004 | 0.008 | 0.029 | 0.008 |  | 0.021* | 0.735 | 0.159* | |
| *RF39* | 0.004 | 0.021 | 0.005 | 0 |  | 1.000 | 0.124* | 0.074* | |
| *Paeniclostridium* | 0 | 0 | 0.015 | 0.008 |  | 0.030* | 1.000 | 0.646 | |
| *Proteus* | 0.013 | 0.001 | 0.008 | 0.003 |  | 0.735 | 0.225* | 0.772 | |
| *Clostridioides* | 0 | 0.004 | 0.012 | 0 |  | 0.067* | 0.655 | 0.095* | |
| *Sphingomonas* | 0.012 | 0 | 0.005 | 0 |  | 0.494 | 0.198* | 0.735 | |
| *Gordonibacter* | 0.009 | 0 | 0.004 | 0.002 |  | 0.421 | 0.092* | 0.474 | |
| *Butyricicoccus* | 0 | 0 | 0.008 | 0.006 |  | 0.241* | 1.000 | 0.932 | |
| *Woeseia* | 0 | 0 | 0 | 0.017 |  | 1.000 | 1.000 | 0.159* | |
| *Prevotellaceae_UCG-001* | 0.002 | 0.002 | 0.002 | 0.009 |  | 1.000 | 1.000 | 0.241* | |
| *Hungatella* | 0.010 | 0 | 0 | 0 |  | 0.067* | 0.095* | 0.259 | |
| *Christensenellaceae* | 0 | 0 | 0 | 0.007 |  | 1.000 | 1.000 | 0.222* | |
| *Erysipelatoclostridiaceae* | 0.003 | 0 | 0.001 | 0 |  | 0.272 | 0.208* | 0.544 | |

**q*-value < 0.25. Statistical analysis by MaAsLin2.


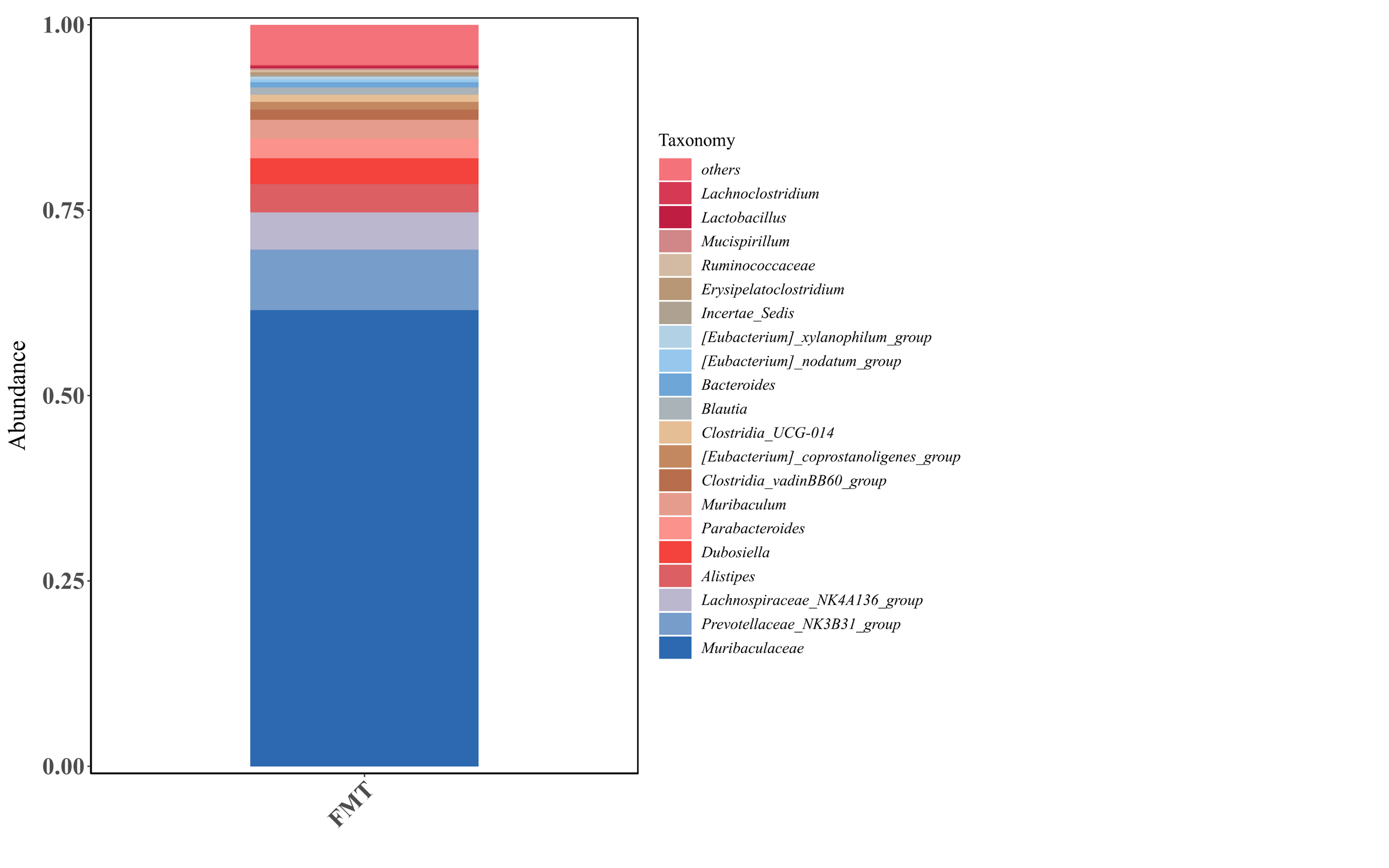


Figure S1. The microbial composition of the FMT suspension at the genus level


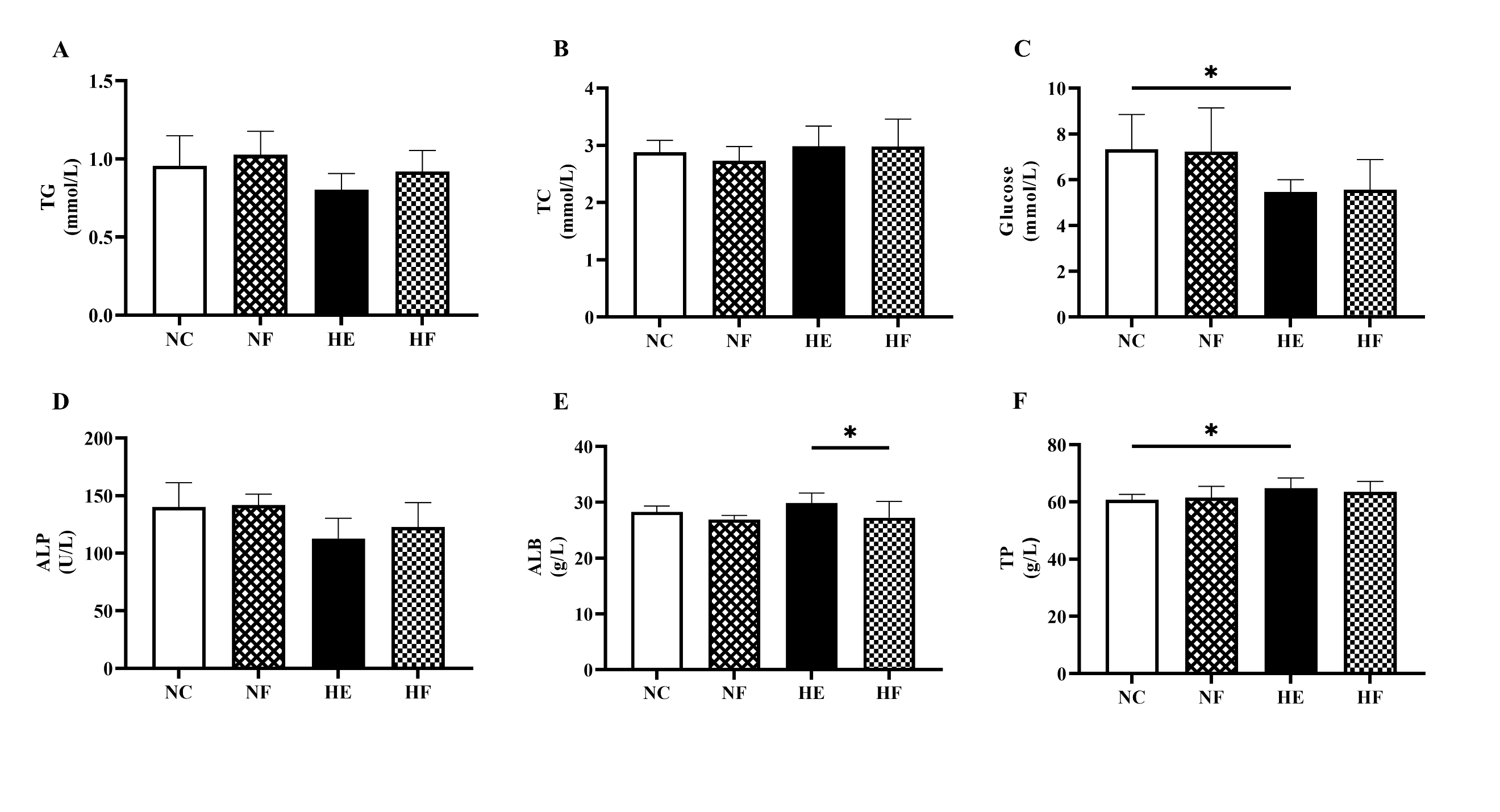


Figure. S2. Circulating biochemical measurements. Serum levels of TG (A), TC (B), glucose (C), ALP (D), ALB (E), and TP (F). Groups: NC, normal control (22℃ only); NF, 22℃ + FMT; HE, heat exposure (39℃ only); HF: 39℃ + FMT; n = 6 per group. Statistical analysis was performed using two-way ANOVA. Statistical analysis was performed using two-way ANOVA. **P* < 0.05.


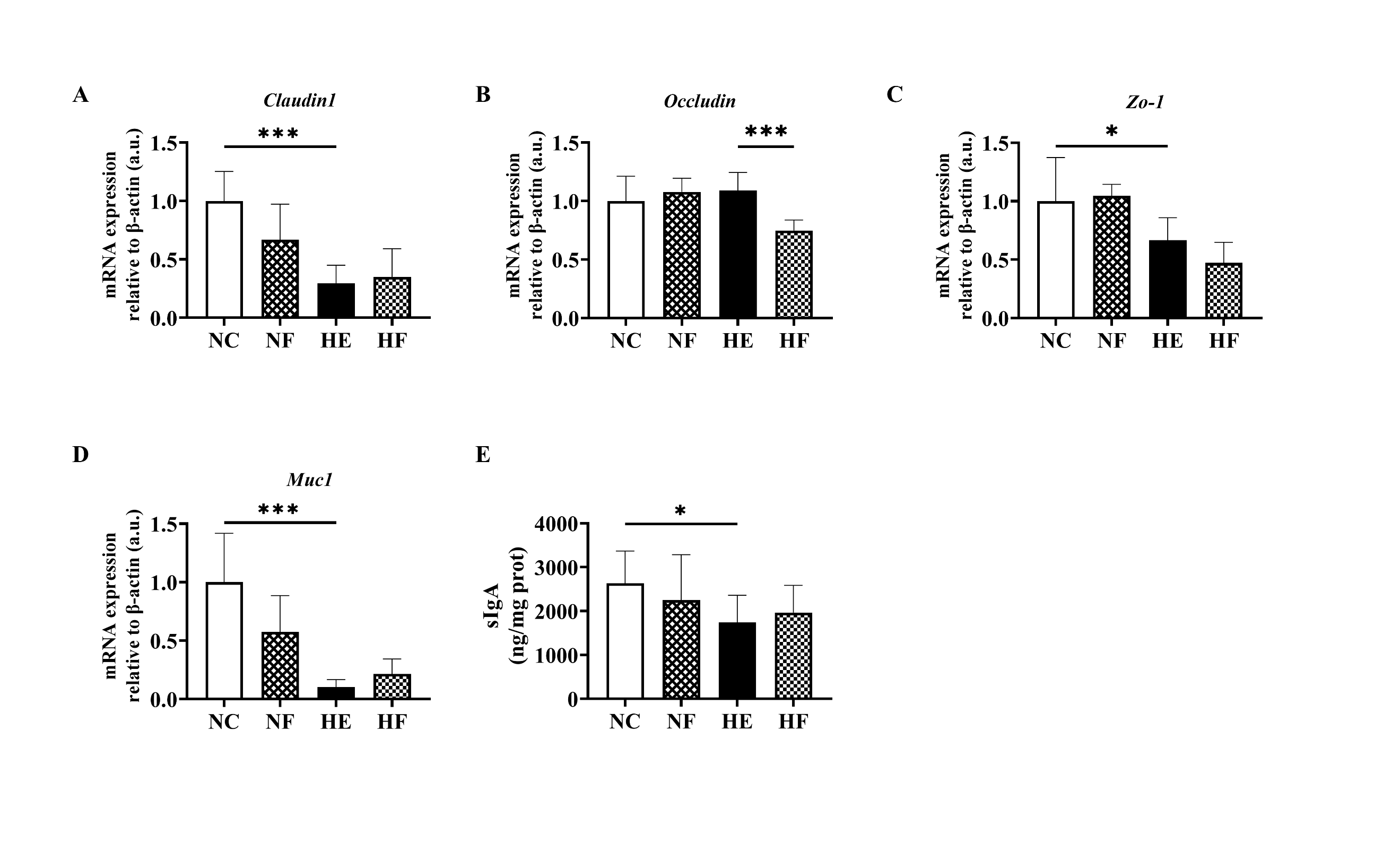


Figure. S3. Markers of colonic barrier integrity and mucosal immunity. Relative mRNA expression levels of *Claudin1* (A), *Occludin* (B), *Zo-1* (C), *Muc1* (D), and secretory IgA (sIgA) levels (E) in the colon. Groups: NC, normal control (22℃ only); NF, 22℃ + FMT; HE, heat exposure (39℃ only); HF, 39℃ + FMT; n = 6 per group. Statistical analysis was performed using two-way ANOVA. **P* < 0.05, and ****P* < 0.001.


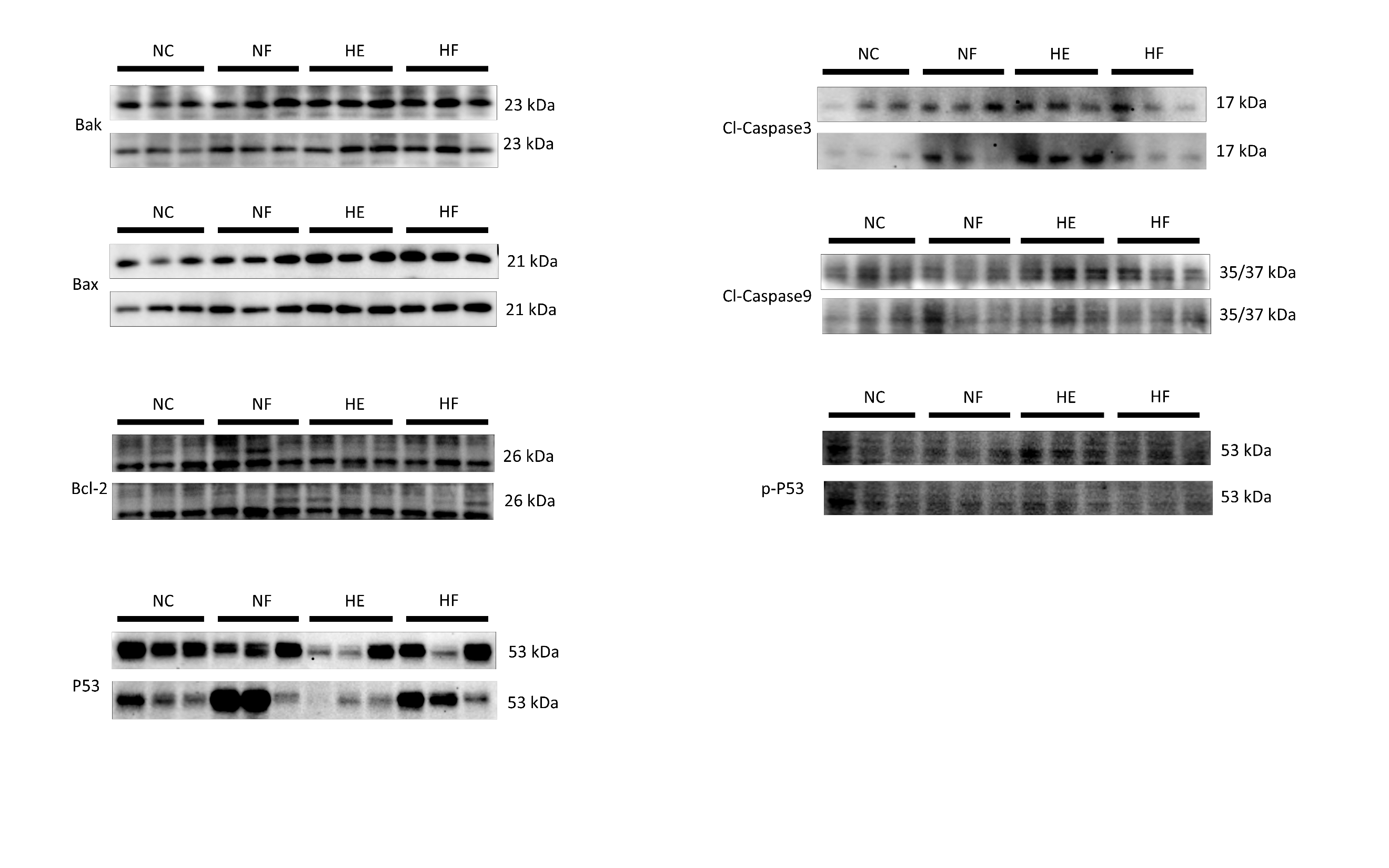


Figure S4. Complete protein gel images for all samples.
